# Exosomes derived from miR-26a-5p-modified adipose mesenchymal stem cells improve wound healing by targeting *MAP2K4*

**DOI:** 10.3389/fbioe.2025.1662095

**Published:** 2025-11-11

**Authors:** Kana Chen, Wei Ye, Longjun Chi, Shujie Xie

**Affiliations:** 1 Department of Plastic Surgery, Ningbo NO. 2 Hospital, Ningbo, Zhejiang, China; 2 Department of Hepatobiliary and Pancreatic Surgery, Ningbo NO. 2 Hospital, Ningbo, Zhejiang, China

**Keywords:** AMSCs-derived exosomes, miR-26a-5p, MAP2K4, angiogenesis, wound healing

## Abstract

**Introduction:**

Abnormal wound healing impairs bodily functions and burdens healthcare systems. Adipose mesenchymal stem cells (AMSCs)-derived exosomes promote wound healing, with exosomal microRNAs (miRNAs) playing pivotal roles. This study investigated the roles and mechanisms of miR-26a-5p (delivered by AMSCs-derived exosomes) in wound healing.

**Methods:**

The GSE55661 dataset was analyzed to screen a crucial miRNA (miR-26a-5p) and its target gene (*MAP2K4*), and their interaction was further validated by dual-luciferase reporter gene assay. Exosomes were isolated from miR-26a-5p-overexpressing AMSCs, and a mouse skin defect model was used to evaluate their effects on wound healing.

**Results:**

Bioinformatics identified 13 differentially expressed miRNAs, and a miRNA-mRNA regulatory network composed of 12 DEmiRNAs and 143 regulated target genes was built. In this network, miR-26a served as the hub node, and the target genes were enriched in the MAPK cascade, as well as cAMP, relaxin, Hippo, Apelin, Wnt, and cGMP-PKG signaling pathways. Thereafter, *MAP2K4* was identified as the target of miR-26a-5p, and exosomes were successfully isolated from AMSCs overexpressing miR-26a-5p. Exosomes from miR-26a-5p overexpressed AMSCs (like miR-26a-5p agomir) could facilitate wound healing, and down-regulated *MAP2K4*, *Il6*, *Il1β*, and *Tnf-α*, whereas up-regulated *Col1a1*, *Cd31*, *Col2a1*, *α-Sma*, and *Col3a1*.

**Discussion:**

AMSCs-derived exosomes delivering miR-26a-5p may expedite wound healing by targeting MAP2K4, inhibiting inflammation, and enhancing angiogenesis and ECM synthesis.

## 1 Introduction

Wound healing, a complex and dynamic physiological process, begins immediately after injury and continues for months or years after wound closure, involving multiple stages such as the inflammatory response, cell proliferation, angiogenesis, and extracellular matrix (ECM) remodeling (Hassanshahi et al., 2022; Sorg and Sorg, 2023). During the inflammatory phase, it lays the foundation for subsequent repair by clearing pathogens and necrotic tissues (Hassanshahi et al., 2022). During the proliferation phase, granulation tissues and neovascularization are formed to provide support for wound filling and nutrient supply (Sharifiaghdam et al., 2022). During the remodeling period, the newly formed tissues are refined to resemble normal tissues in both structure and function (Al-Masawa et al., 2022). Generally, wounds are classified into acute and chronic wounds. Acute wounds typically heal at a predictable and expected rate, whereas wounds that fail to heal within 6 weeks and exhibit inefficient cellular and molecular function are classified as chronic wounds (Dai et al., 2020). If not properly treated, they may increase the incidence rate and medical care costs of patients, and even lead to amputation in serious cases (Cioce et al., 2024). At present, the methods used in clinical practice to promote wound healing include various types of wound dressings, drug therapy (such as antibiotics, growth factors), physical therapy (such as negative pressure wound therapy, laser therapy), and skin transplantation (Wilkinson and Hardman, 2020; Freedman et al., 2023). However, although these approaches may exhibit some benefits, their widespread use is limited by the emergence of drug resistance and immune rejection reactions, complex administration methods, and high costs (Kolimi et al., 2022). Therefore, an in-depth understanding of the underlying mechanisms of wound healing, along with the development of more effective new therapeutic strategies to promote wound healing, is essential in the field of regenerative medicine.

Mesenchymal stem cells (MSCs), including adipose mesenchymal stem cells (AMSCs), are multipotent stem cells with self-renewal ability, multipotent differentiation potential, and paracrine regulation (Zhou et al., 2023). Due to their ease of isolation, *in vitro* expansion, and multipotency, AMSCs have been recognized as a crucial source of stem cells in the field of regenerative medicine, including tissue repair and regeneration (Guillamat-Prats, 2021). Notably, the therapeutic utilization of stem cells in wound healing is also limited by storage challenges, mutation-related tumorigenicity, optimal cell activity, immune rejection, and ethical factors (Margiana et al., 2022). Previous research has reported that AMSCs can activate a series of bioactive factors through autocrine and paracrine pathways, thereby participating in the healing process of skin injuries (Mazini et al., 2020). Exosomes, with a size of approximately 30–150 nm, can be secreted by almost all cells and absorbed by cells through autocrine or paracrine pathways, which are the primary contributors to stem cell efficacy (Tan et al., 2024). Zhou et al. (2022) demonstrated that hydrogel Pluronic F-127 (PF-127) loaded in the AMSCs-derived exosomes could improve the efficiency of exosome delivery, and keep the bioactivity of AMSCs-derived exosomes; as well as have a better effect on promoting wound healing and regeneration than AMSCs-derived exosomes administered alone. Another study prepared a biological scaffold of AMSCs-derived exosomes modified gelatin sponge/polydopamine scaffold (GS-PDA-Exos), and found that AMSCs-derived exosomes could be released slowly from GS-PDA-Exos. The study also showed that GS-PDA-Exos had significant potential in the treatment of bone defects (Li et al., 2023). Furthermore, AMSCs-derived exosomes can promote skin wound healing by influencing all stages of wound healing, including regulating inflammatory responses, promoting the proliferation and migration of fibroblasts and keratinocytes, promoting angiogenesis, and regulating extracellular matrix remodeling (Weiliang and Lili, 2021). These investigations further confirmed that AMSCs-derived exosomes are a very promising and novel factor in wound repair and regeneration.

Exosomes are rich in proteins, lipids, microRNAs (miRNAs), long non-coding RNAs (lncRNAs), and mRNAs; they are considered a communication tool between cells and a promising biological gene delivery system. Exosomes can endow miRNAs with considerable stability and resistance to RNA enzyme degradation, thereby exerting post-transcriptional gene regulation through their miRNA content. MiRNAs, a small endogenous RNA molecule with a length of approximately 22 nucleotides (nt), have been shown to play a crucial role in health and disease, including various types of cancer, cardiovascular disease, and wound healing. Wang et al. (2021) used high-throughput sequencing to demonstrate that upregulated miR-126-5p, miR-21-3p, and miR-31-5p, while downregulated miR-99b and miR-146a, were associated with wound healing. Additionally, hypoxic AMSCs-derived exosomes were found to promote diabetic wound healing and inhibit inflammation through the PI3K/AKT signaling pathway. Another investigation revealed that the level of miR-488-3p was significantly decreased in the wound tissues of diabetic patients with skin defects compared to the control group, and miR-488-3p overexpression could accelerate wound healing by targeting the MeCP2 and CYP1B1-mediated Wnt/β-catenin signaling pathway (Zuo et al., 2024). Therefore, miRNAs play crucial roles in regulating various biological processes, including wound healing. Additionally, the roles of miR-26a-5p have been reported in many cancers (Cai et al., 2022; Zhang et al., 2023); however, its potential roles and underlying mechanisms, particularly with AMSCs-derived exosomes as carriers in wound healing, need to be further explored.

In our study, miRNA profiles associated with wound injury were downloaded from NCBI Gene Expression Omnibus (GEO), and a series of bioinformatics analyses were performed to identify the crucial miRNAs. Based on the regulatory network, miR-26a-5p served as the hub node, and its target gene, MAP2K4, participated in the MAPK cascade, relaxin signaling pathway, and growth hormone synthesis, secretion, and action. Therefore, the interaction between miR-26a-5p and *MAP2K4* was further validated, and the roles and related potential mechanisms of AMSCs-derived exosomal miR-26a-5p in wound healing were investigated *in vivo*. Notably, while miR-26a-5p has been reported to exert regenerative effects in other tissues, such as inhibiting epithelial-mesenchymal transition (EMT) in lung fibrosis via human umbilical cord MSC (hUCMSC)-derived exosomes (Zhao et al., 2023) and alleviating inflammation in diabetic retinopathy through the USP14/NF-κB pathway (Bian et al., 2024), but its role in cutaneous wound healing remains unaddressed. Two critical gaps distinguish our work from these prior studies: first, the tissue-specific regulatory mechanisms of miR-26a-5p differ across organs—lung fibrosis and retinal inflammation involve distinct cell types (e.g., alveolar epithelial cells, retinal Müller cells) and pathways (e.g., EMT, NF-κB) that are not the core drivers of skin wound healing (which relies on inflammation resolution, angiogenesis, and extracellular matrix (ECM) synthesis). Second, prior studies have utilized non-adipose MSC sources (e.g., hUCMSCs) or administered miRNA directly. In contrast, we focus on AMSCs-derived exosomes, a carrier with unique advantages for skin repair, including easier isolation from adipose tissue, stronger paracrine potential for activating cutaneous cells, and better biocompatibility with the skin microenvironment. Moreover, our bioinformatics analysis identifies MAP2K4 (a key upstream regulator of the MAPK cascade) as a novel target of miR-26a-5p in wound healing, which is not reported in lung or retinal studies. This tissue-specific target and carrier system combination fills the gap in understanding miR-26a-5p′s role in skin repair and provides a more translational strategy for wound therapy.

## 2 Methods

### 2.1 Bioinformatics analysis

The expression profile of GSE55661 was downloaded from NCBI GEO (http://www.ncbi.nlm.nih.gov/geo/) (Edgar et al., 2002), which contained miRNA expression profile data from 18 mouse skin tissue samples. The tested platform was GPL18386 Luminex Multi-species miRNA array (miRBase 8.0). Then, we selected 6 samples, 2 days after the injury, including 3 samples from the damaged area and 3 normal control skin samples. The limma package version 3.34.7 in R4.3.1 (https://bioconductor.org/packages/release/bioc/html/limma.html) (Ritchie et al., 2015) was used to identify the differentially expressed miRNAs (DEmiRNAs) between the damaged samples and normal control samples based on the thresholds of false discovery rate (FDR) < 0.05 using the Benjamini and Hochberg method and |log_2_ fold change (FC)| > 1.

Thereafter, the identified DEmiRNAs were submitted for the search of target genes using the miRWalk 3.0 database (http://mirwalk.umm.uni-heidelberg.de/) (Hemmat et al., 2020). The linkage pairs (miRNA-mRNA pairs) labeled with “validated” (i.e., regulatory linkage confirmed by experiments) were retained and visualized using Cytoscape version 3.6.1 (http://www.cytoscape.org/) (Shannon et al., 2003). After that, the target genes in the network were subjected to the functional analyses based on DAVID version 6.8 (https://david.ncifcrf.gov/), including biological process (BP) of gene ontology (GO) terms and Kyoto Encyclopedia of Genes and Genomes (KEGG) pathways; as well as FDR <0.05 with the Benjamini and Hochberg method was selected as the threshold of enrichment significance.

### 2.2 Dual-luciferase reporter gene assay

The wild-type and mutant sequences of MAP2K4 3′-untranslated region (3′-UTR) were synthesized, and the pGL3-basic vector (Yanzai Biotechnology, Shanghai, China) was employed to construct the 3′-UTR MAP2K4 report plasmids (pGL3-MAP2K4-WT and pGL3-MAP2K4-MUT). Thereafter, the pGL3-basic vector (500 ng), pGL3-MAP2K4-WT (500 ng) and pGL3-MAP2K4-MUT (500 ng) were co-transfected with 293T cells (Cell Bank. Chinese Academy of Science, Shanghai, China) with miR-26a-5p mimics (100 nM) or negative control (NC) mimics (100 nM) using Lipofectamine 2000 (Thermo Fisher Scientific, United States) in line with the manufacturer’s protocols. After transfection for 6 h, the medium was changed to complete medium, and the cells were cultured for another 48 h. After that, the cells were harvested to determine the relative luciferase activity using a dual luciferase reporter system (Promega, WI, United States) according to the manufacturer’s recommendations.

### 2.3 Cell culture and transfection

Mouse AMSCs were purchased from Cyagen Biosciences Inc. (Guangzhou, China) and were maintained in α-MEM medium (Servicebio, Wuhan, China) supplemented with 10% fetal bovine serum (FBS, Thermo Fisher Scientific, United States) and 1% penicillin/streptomycin (Thermo Fisher Scientific). The construction of AMSCs with miR-26a-5p overexpression was performed using Lipofectamine 200 (Thermo Fisher Scientific) based on the manufacturer’s instructions. Briefly, the mouse AMSCs were seeded into a 24-well plate at a density of 4 × 10^4^, and cultured overnight. The next day, the cell medium was changed to serum-free medium, and the cells were transfected with 15 pmol miR-26a-5p agomir (GeneRay, Shanghai, China) or NC (GeneRay) using Lipofectamine 200. After 6 h of transfection, the medium was replaced with complete medium. After culturing for an additional 48 h, total RNA was extracted from the different cells, and the miR-26a-5p level was determined using real-time quantitative PCR (RT-qPCR) to assess the cell transfection efficiency.

### 2.4 Isolation and characterization of AMSCs-derived exosomes

The supernatants of mouse AMSCs and AMSCs transfected with miR-26a-5p agomir were harvested for exosome isolation, and the isolation was performed at 4 °C. The harvested cell supernatants were centrifuged at 500 × g for 5 min, and then the supernatants were transferred to a new tube. After centrifugation at 2000 × g for 30 min, followed by 10,000 × g for 60 min, the supernatant was filtered through a 0.22 μm sterile filter and collected into an ultra-high-speed centrifuge tube. After centrifugation at 120,000 × g for 70 min, the supernatants were removed, and the sediments were resuspended in sterile PBS (200 μL), which contained the exosomes, and were stored at −80 °C.

The concentrations of the isolated exosomes were determined using a BCA assay kit (Beyotime Biotechnology, Shanghai, China) according to the manufacturer’s protocols. Then, a Nanosight NS300 particle size analyzer (NTA; Malvern Panalytical, Malvern, United Kingdom) was used to determine the exosome size distribution based on the method of Yang et al. (2021). The morphology and ultrastructure of isolated exosomes were visualized using a transmission electron microscope (TEM, JEOL LTD, Peabody, MA, United States) as previously reported (Lin et al., 2024). Additionally, the expression of exosome-specific proteins, including CD63, CD81, and HSP70, and a negative control protein (calnexin), was detected by Western blot using their corresponding antibodies.

### 2.5 Animal experiments

A total of 24 specific pathogen-free (SPF) male C57BL/6 mice weighing 20 ± 2 g were purchased from Shanghai SLAC Laboratory Animal Co., Ltd (Shanghai, China). All the mice were kept under controlled temperature (24 °C ± 2 °C) and humidity (50% ± 5%), with a 12 h light/dark cycle. All the mice had free access to standard laboratory food and filtered water during the experiments. Our animal experiments were conducted in strict accordance with the guidelines and regulations of the Institutional Animal Care and Use Committee (IACUC) of Guoke Ningbo Life Science and Health Industry Research Institute (approval no. GX-2025-XM-0004). The committee reviewed and approved the experimental protocol to ensure the ethical treatment of animals.

After 7 days of acclimatization, all the mice were randomly divided into four groups (n = 6 for each group): control, model, AMSCs-agomir-Exo, and miR-26a-5p agomir groups. The mice in the model, AMSCs-agomir-Exo and miR-26a-5p agomir groups, were first used to establish a skin defect mouse model as previously reported (Liu et al., 2023). Briefly, the mice were deeply anesthetized using 4% isoflurane, and then fixed in a prone position. Thereafter, the back hair of the mice was removed, and the area was disinfected with 75% alcohol. A circular full-thickness skin defect with a diameter of 2 cm was then created. On the first day after injury, the mice in the model, AMSCs-agomir-Exo, and agomir groups were injected with PBS, exosomes isolated from AMSCs with miR-26a-5p overexpression (AMSCs-agomir-Exo, 200 μg/mice), and miR-26a-5p agomir (20 nmol/mice), respectively. The mice in the control group did not receive any treatment. Additionally, 12 C57BL/6 mice were obtained and randomly divided into two groups (n = 6 for each group): the si-NC and si-MAP2K4 groups. Firstly, all the mice were used to construct a skin defect mouse model. The mice in the si-NC and si-MAP2K4 groups were injected with si-NC (20 nmol/mouse) and si-MAP2K4 (20 nmol/mouse), respectively, on the first day after injury. After treatment for 0, 4, 8, and 12 days, the wounds of each mouse in the different groups were photographed and compared. Using a 2 cm × 2 cm unified field of view, image analysis software IPP 6.0 was used to analyze the change of wound area. Additionally, on the 12th day after treatment, all mice were sacrificed by cervical dislocation, and the wounds and surrounding skin tissues were collected. A portion of the tissue was fixed with 4% paraformaldehyde for subsequent histopathological analysis, and the other part was used for further RT-qPCR and Western blot analysis.

### 2.6 Histopathological analysis

The fixed tissue samples were dehydrated, embedded in paraffin, and cut into 4-μm slices. After baking at 60 °C for 30 min, the slices were dewaxed and rehydrated. For hematoxylin-eosin (HE) staining, the processed slices were stained with hematoxylin for 10 min, and re-stained with eosin for 90 s. After dehydration, transparentization, and sealing, the slices were scanned and photographed under an optical microscope (Olympus Corporation, Tokyo, Japan).

For Masson staining, the slices were treated with potassium dichromate mordant for 12 h, and after washing, they were stained with Weigert iron hematoxylin for 10 min. After washing and differentiation with 1% hydrochloric acid alcohol, the slices were stained with Lichun red acid fuchsin dye for 10 min. After washing and being treated with phosphomolybdic acid for 150 s, the slices were re-stained with aniline blue dye for 5 min. After being treated with 1% glacial acetic acid for 1 min and dehydrated with ethanol, the slices were sealed with neutral gum, and the images were observed using an optical microscope (Olympus Corporation).

### 2.7 Real-time quantitative PCR (RT-qPCR)

Total RNA was isolated using an RNA extraction solution (Servicebio) according to the manufacturer’s instructions, and then qualified and quantified using a microplate reader. Afterward, total RNA (1 μg) was reverse transcribed into cDNA using the PrimeScriptTM II 1st Strand cDNA synthesis kit (TaKaRa, Osaka, Japan), according to the manufacturer’s instructions. The sequences of all primers are displayed in Table 1.

**TABLE 1:** The sequences of all primers.

Primer	Sequence (5′-3′)
*Tnf-α*	mF: CTG​AAC​TTC​GGG​GTG​ATC​GG
	mR: GGC​TTG​TCA​CTC​GAA​TTT​TGA​GA
*Il6*	mF: TAG​TCC​TTC​CTA​CCC​CAA​TTT​CC
	mR: TTG​GTC​CTT​AGC​CAC​TCC​TTC
*Il1β*	mF: TGC​CAC​CTT​TTG​ACA​GTG​ATG
	mR: TGA​TGT​GCT​GCT​GCG​AGA​TT
*α-Sma*	mF: CCC​AGA​CAT​CAG​GGA​GTA​ATG​G
	mR: TCT​ATC​GGA​TAC​TTC​AGC​GTC​A
*Mapk10*	mF: AGG​TGG​ACA​ACC​AGT​TCT​ACA
	mR: GCA​CAG​ACT​ATT​CCC​TGA​GCC
*Col3a1*	mF: CAA​AGT​GGA​ACC​TGG​TTT​CTT​C
	mR: TCT​AGT​GGC​TCC​TCA​TCA​CAG​A
*Col1a1*	mF: GCT​CCT​CTT​AGG​GGC​CAC​T
	mR: CCA​CGT​CTC​ACC​ATT​GGG​G
*Col2a1*	mF: CAG​TAC​CTT​GAG​ACA​GCA​CGA​C
	mR: CAG​TAG​TCT​CCG​CTC​TTC​CAC​T
*Cd31*	mF: GAC​CCA​GCA​ACA​TTC​ACA​GAT​A
	mR: TCT​TTC​ACA​GAG​CAC​CGA​AGT​A
*Gapdh*	mF: GGT​GAA​GGT​CGG​TGT​GAA​CG
	mR: CTC​GCT​CCT​GGA​AGA​TGG​TG
miR-26a-5p	RT: GTCGTATCCAGTGCAGGGTCCGAGGTA TTCGCACTGGATACGACTGGGGT
	F: TGTCAGTTTGTCAAAT
U6	F: CTCGCTTCGGCAGCACA
	R: AAC​GCT​TCA​CGA​ATT​TGC​GT
Downstream universal primer	GTGCAGGGTCCGAGGT

For the miR-26a-5p level, the stem loop RT-PCR method was employed with U6 as the endogenous reference gene. First, a 10 μL denaturation mixture was prepared for miR-26a-5p reverse transcription. The subsequent reverse transcription reaction was carried out in a 20 μL total volume, containing 10 μL of denaturation solution, 4 μL of 5X PrimeScript II buffer, 0.5 μL of RNase inhibitor (40 U/μL), 1 μL of PrimeScript II reverse transcriptase (200 U/μL), and 4.5 μL of RNase-free distilled water. The reverse transcription procedure consisted of incubation at 42 °C for 60 min, followed by a 5-min incubation at 95 °C to inactivate the enzyme. For qPCR, the thermal profile began with an initial denaturation step at 95 °C for 5 min, followed by 40 cycles of 95 °C for 10 s and 60 °C for 30 s.

For the expression levels of related genes, *Gapdh* served as a housekeeping gene. The thermal cycling protocol used for the associated genes reserve transcription involved incubation of the reverse transcription mix at 37 °C for 60 min and 85 °C for 5 s. The RT-qPCR reaction was initiated at 95 °C for 30 s, followed by a total of 40 cycles at 95 °C for 5 s and 60 °C for 30 s, and then a melting step at 95 °C for 15 s, 60 °C for 60 s, and 95 °C for 15 s. Finally, the miR-26a-5p level and the expression of *Map2k4*, *Col1a1*, *Col2a1*, *Col3a1*, *α-Sma*, *Tnf-α*, *Il1β*, *Il6*, and *Cd31* were analyzed using the 2^−ΔΔCT^ method (Xu et al., 2019).

### 2.8 Western blotting

Total proteins were extracted from tissue samples or exosome samples using RIPA (Beyotime Biotechnology) and were quantified by a BCA assay kit (Beyotime Biotechnology). Subsequently, the total protein samples were separated by 10% SDS-PAGE (stacking gel: 60 V for 30 min; and running gel: 110 V for 120 min), and then transferred to PVDF membranes (100 V for 50 min on ice). After blocking with 5% skim milk at 37 °C for 2 h, the membranes were incubated the primary antibodies, including anti-CD63 antibody (1: 1,000, Abclonal, Wuhan, China), anti-CD81 antibody (1: 1,000, Abclonal), anti-HSP70 antibody (1: 1,000, Proteintech, Wuhan, China), anti-calnexin antibody (1: 1,000, Proteintech), anti-MAP2K4 antibody (1: 1,000, Proteintech), anti-COL1A1 antibody (1: 1,000, NOVUS, St. Louis, MO, United States), anti-α-SMA antibody (1: 1,000, Cell Signaling Technology, Boston, MA, United States), anti-TNF-α antibody (1: 1,000, Cell Signaling Technology), and anti-GAPDH antibody (1: 10,000, Proteintech). After incubation overnight at 4 °C, the membranes were incubated with the secondary antibodies ((H + L)-HRP goat anti-rabbit/anti-mouse IgG) at 37 °C for 2 h. After washing, the protein bands were visualized using a Millipore ECL kit (Beyotime Biotechnology) and photographed using a system (Shanghai Tanon Technology Co., Ltd., Shanghai, China).

### 2.9 Statistical analysis

Each experiment was repeated three times, and data were reported as mean ± standard deviation. The statistical analyses were conducted using the SPSS software, and all figures were plotted in GraphPad Prism 9 (GraphPad software, San Diego, CA, United States). Before the analyses, a homogeneity of variance test was performed on all data, and the P values were obtained. For comparisons between two independent groups, the independent-samples t-test was used. For comparisons among more than two groups, if the P value > 0.05, a one-way analysis of variance (ANOVA) followed by Least-Significant Difference (LSD) was applied for multiple comparisons; if the P value ≤0.05, ANOVA followed by Dunnett T3 was employed. Statistical significances were set at a *P*-value of less than 0.05.

## 3 Results

### 3.1 Identification of DEmiRNAs and their functional analyses

**FIGURE 1:**
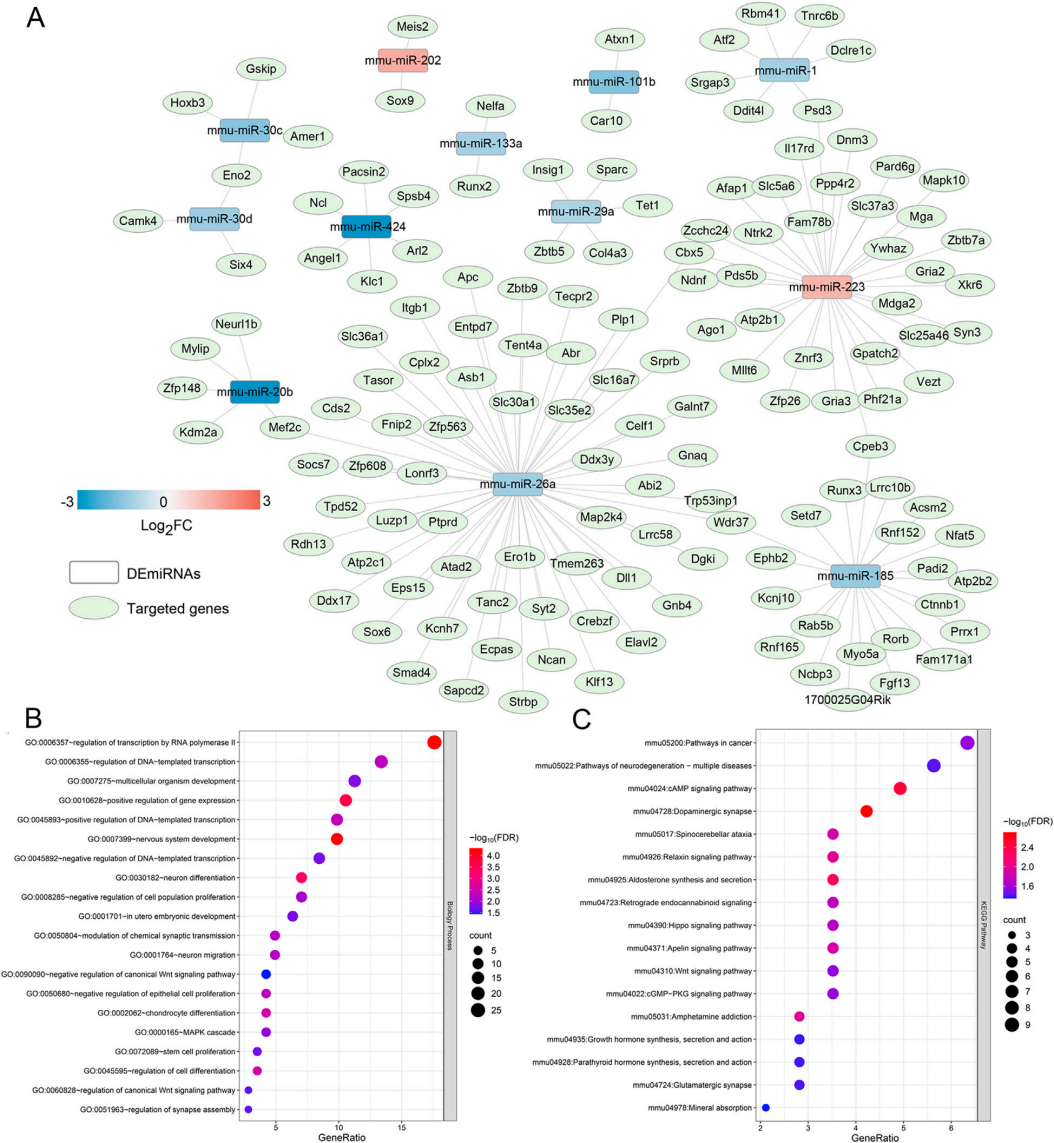
Construction of a miRNA-mRNA regulatory network, and functional analysis of the genes in the network. **(A)** The miRNA-mRNA regulatory network composed of 12 differentially expressed miRNAs and 143 regulated target genes, and miR-26a, which was down-regulated in the damaged tissues, was the hub node. **(B)** The significant gene ontology terms of biological process enriched by the genes in the network. **(C)** The significant Kyoto Encyclopedia of Genes and Genomes pathways enriched by the target genes in the network.

Based on FDR <0.05 and |log_2_FC| > 1, 13 DEmiRNAs were screened between the damaged tissue samples and normal control samples, including miR-424, miR-20b, miR-101b, miR-30c, miR-185, miR-30d, miR-26a, miR-133a, miR-26b, miR-1, miR-29a, miR-223, and miR-202 (Table 2). Then, these DEmiRNAs were submitted for the search of target genes. We identified 149 miRNA-mRNA regulatory pairs labeled as “validated,” and a miRNA-mRNA regulatory network composed of 12 DEmiRNAs and 143 regulated target genes was constructed (Figure 1A). In this network, miR-26a, which was downregulated in the damaged tissues, served as the hub node and was chosen for further experiments.

**TABLE 2:** Identification of differentially expressed miRNAs (DEmiRNAs) between the damaged samples and normal control samples.

DEmiRNAs	Log_2_fold change	P. Value
mmu-miR-424	−25.14641839	1.62E-07
mmu-miR-20b	−23.9947469	9.42E-06
mmu-miR-101b	−1.54202333	0.016052407
mmu-miR-30c	−1.522044653	0.014723127
mmu-miR-185	−1.30294479	0.035499402
mmu-miR-30d	−1.24600513	0.000566504
mmu-miR-26a	−1.220513853	0.012598226
mmu-miR-133a	−1.14807093	0.004639082
mmu-miR-26b	−1.09743327	0.007920801
mmu-miR-1	−1.060731817	0.006365412
mmu-miR-29a	−1.037504953	0.040693745
mmu-miR-223	1.285371387	0.008117965
mmu-miR-202	1.480981323	0.049105714

After that, the genes in the built network were used for BP of GO terms and KEGG analyses. It was found that these genes were significantly enriched in 21 GO terms of BP, and 17 KEGG pathways. The significantly GO terms of BP contained “MAPK cascade”, “regulation of transcription nu RNA polymerase II”, “multicellular organism development”, “neuron differentiation”, “negative regulation of canonical Wnt signaling pathway”, “chondrocyte differentiation”, and “stem cell proliferation” (Figure 1B). Additionally, these genes were also closely associated with “cAMP signaling pathway”, “relaxin signaling pathway”, “Hippo signaling pathway”, “Apelin signaling pathway”, “Wnt signaling pathway”, “cGMP-PKG signaling pathway”, and “glutamatergic synapse” (Figure 1C).

### 3.2 MAP2K4 as a target of miR-26a-5p

**FIGURE 2:**
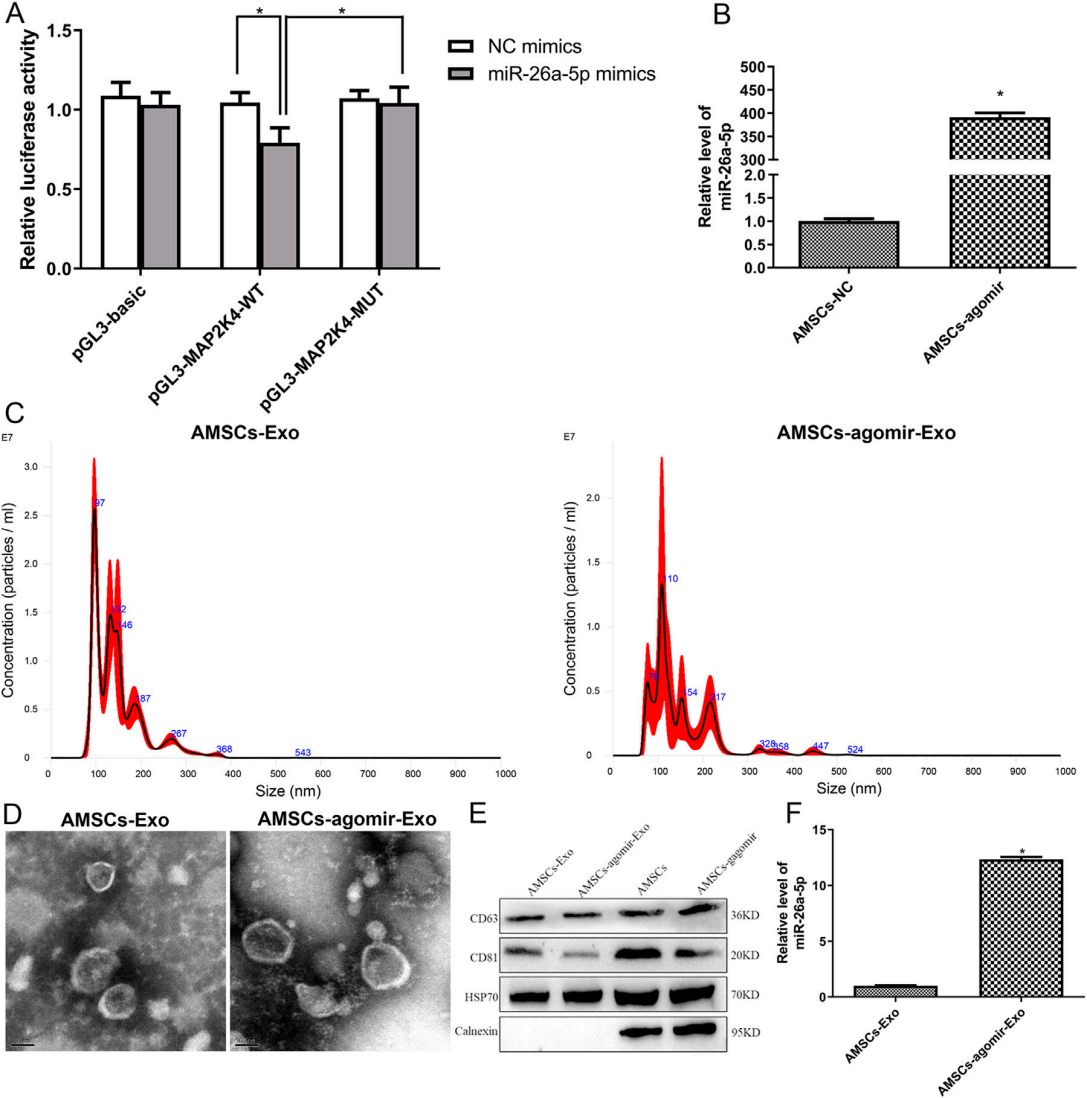
Interaction between miR-26a-5p and *MAP2K4*, as well as cell transfection efficiency and characterization of the isolated exosomes. **(A)** *MAP2K4* was the target of miR-26a-5p by dual luciferase reporter gene assay. N = 3. *: *P* < 0.05. **(B)** The miR-26a-5p level in the AMSCs with miR-26a-5p overexpression to verify the cell transfection efficiency. N = 3. *: *P* < 0.05, vs. AMSCs-NC. **(C)** Particle size distribution of AMSCs-derived exosomes and miR-26a-5p overexpressed AMSCs-derived exosomes determined by Nanosight. **(D)** The morphology of AMSCs-derived exosomes and miR-26a-5p overexpressed AMSCs-derived exosomes, visualized by transmission electron microscopy. **(E)** The expression of exosomes-specific markers (CD63, CD81, and HSP70) and negative control protein (calnexin) in exosomes and cells, detected by Western blot. **(F)** The level of miR-26a-5p in the AMSCs-derived exosomes and miR-26a-5p overexpressed AMSCs-derived exosomes. N = 3. *: *P* < 0.05, vs. AMSCs-Exo.

Based on the proposed miRNA-mRNA regulatory network, we observed that miR-26a interacted with *MAP2K4*, and *MAP2K4* was significantly involved in the MAPK cascade, relaxin signaling pathway, and growth hormone synthesis, secretion, and action. Therefore, a dual luciferase reporter gene assay was conducted to confirm the relationship between miR-26a-5p and *MAP2K4*. In the pGL3-basic plasmids, there was no significant difference in relative luciferase activity between the NC mimics and miR-26a-5p mimics (*P* > 0.05, Figure 2A). In the pGL3-MAP2K4-WT plasmids, the relative luciferase activity after transfection with miR-26a-5p mimics was evidently lower than that after transfection with NC mimics (*P* < 0.05, Figure 2A). However, when *MAP2K4* was mutated (pGL3-MAP2K4-MUT plasmids), the relative luciferase activity after being transfected with miR-26a-5p mimics was markedly increased in comparison with the pGL3-MAP2K4-WT plasmids transfected with miR-26a-5p mimics (*P* < 0.05, Figure 2A). These outcomes suggested that *MAP2K4* was the target of miR-26a-5p.

### 3.3 Cell transfection efficiency and exosomes characterization

To investigate the effects of AMSCs-derived exosomal miR-26a-5p on wound healing, AMSCs with miR-26a-5p overexpression were constructed with miR-26a-5p agomir, and exosomes were isolated. It was obvious that the levels of miR-26a-5p in the AMSCs and AMSCs transfected with miR-26a-5p agomir were, respectively, 1.00 ± 0.09 and 391.51 ± 16.22, which displayed that the miR-26a-5p level in the AMSCs transfected with miR-26a-5p agomir was significantly higher than that in the AMSCs (*P* < 0.05, Figure 2B). These results revealed that the AMSCs with miR-26a-5p overexpression were successfully constructed and could be employed for subsequent exosome isolation.

Then, the exosomes were isolated from AMSCs and AMSCs with miR-26a-5p overexpression, and were identified by NTA, TEM, and Western blot. Our NTA results showed that the main peaks of the isolated substances from AMSCs and AMSCs with miR-26a-5p overexpression were respectively 96.5 nm and 109 nm, as well as the mean particle sizes of the isolated substances from AMSCs and AMSCs with miR-26a-5p overexpression were 147.4 nm and 151.5 nm, respectively (Figure 2C). The results were in line with the size distribution of exosomes (Krylova and Feng, 2023). Thereafter, the TEM images showed that the isolated substances from AMSCs and AMSCs with miR-26a-5p overexpression exhibited nearly round or cup-shaped morphology with a diameter of approximately 100 nm (Figure 2D). Additionally, Western blot analysis showed that the exosome-specific markers CD63, CD81, and HSP70 were expressed in both the exosomes (AMSCs-derived exosomes and miR-26a-5p overexpressed AMSCs-derived exosomes) and the cells (AMSCs and miR-26a-5p overexpressed AMSCs). However, the expression of negative control protein calnexin was only observed in the cells (Figure 2E). Finally, the level of miR-26a-5p was determined in the AMSC-derived exosomes and in miR-26a-5p-overexpressing AMSC-derived exosomes. Compared with the AMSCs-derived exosomes, the miR-26a-5p level in the miR-26a-5p overexpressed AMSCs-derived exosomes was significantly increased (*P* < 0.05, Figure 2F). These findings indicated that the exosomes were successfully isolated from the miR-26a-5p-overexpressed AMSCs and could be employed in subsequent animal experiments.

### 3.4 Effects of miR-26a-5p overexpressed AMSCs-derived exosomes on wound healing in mice

**FIGURE 3:**
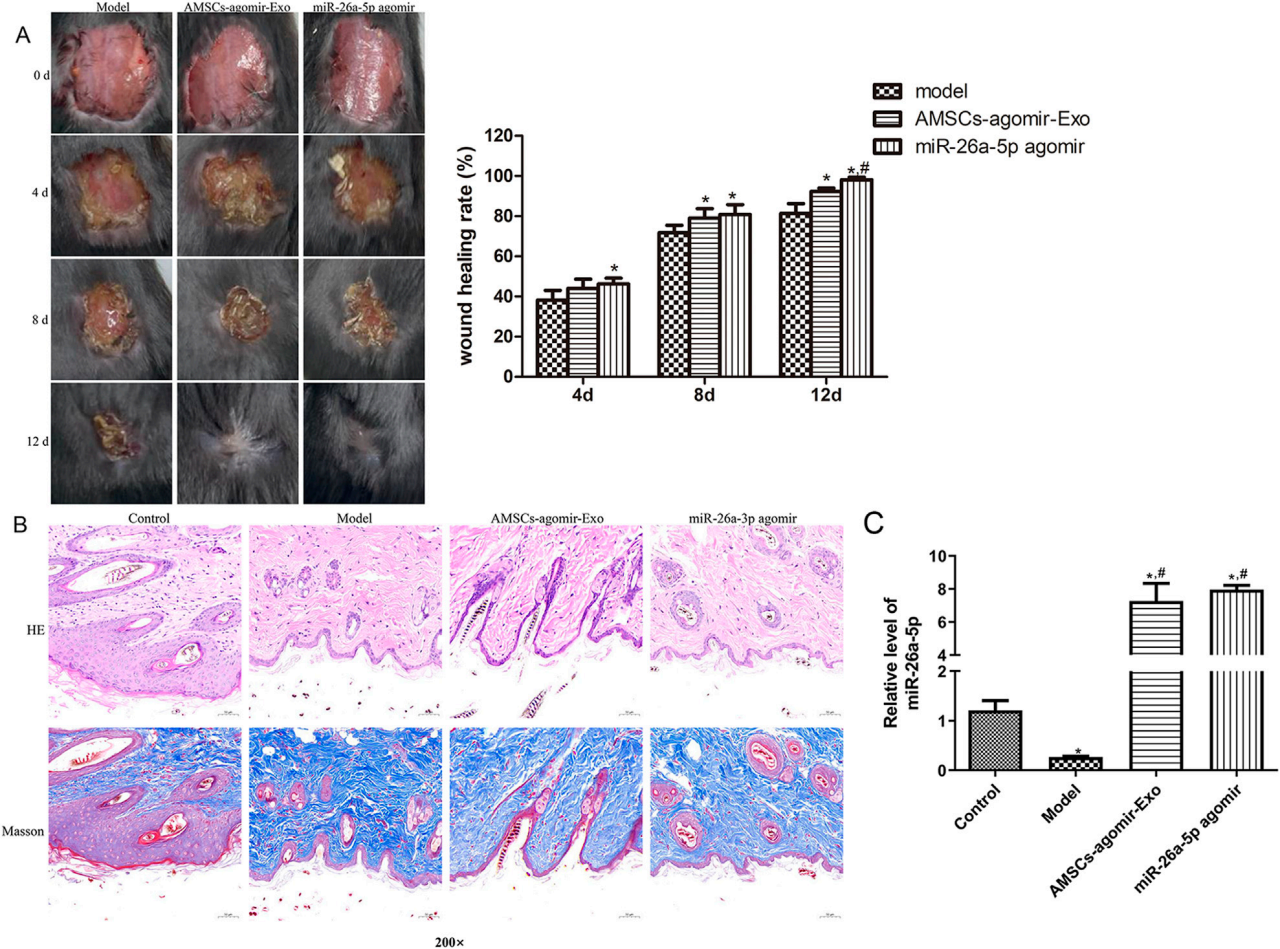
Effects of miR-26a-5p overexpressed AMSCs-derived exosomes on wound healing in mice. **(A)** The wound healing process in the different groups of mice at days 0, 4, 8, and 12. N = 6. *: *P* < 0.05, vs. model; ^#^: *P* < 0.05, vs. AMSCs-agomir-Exo. **(B)** The morphology of the injured skin in the different groups, observed by Hematoxylin-Eosin (HE) staining and Masson staining. N = 6. **(C)** The level of miR-26a-5p in the skin tissues of different mice. N = 3. *: *P* < 0.05, vs. control; ^#^: *P* < 0.05, vs. model.

Further to explore the effects of miR-26a-5p overexpressed AMSCs-derived exosomes on wound healing *in vivo*, a circular full-thickness skin defect with a diameter of 2 cm was created on the back of mice, which were randomized to be administered with miR-26a-5p overexpressed AMSCs-derived exosomes and miR-26a-5p agomir. Wound images were obtained at days 0, 4, 8, and 12. As shown in Figure 3A, the wounds of the mice healed gradually with the gradual increase of the days, as well as the wounds of the mice in the AMSCs-agomir-Exo and miR-26a-5p agomir healed well on day 12 after injury. At day 12, the wound healing rates were significantly elevated in the AMSCs-agomir-Exo and miR-26a-5p agomir groups compared to the model group (*P* < 0.05), and were evidently increased in the AMSCs-agomir-Exo group in comparison with the miR-26a-5p agomir group (*P* < 0.05, Figure 3A). After that, the morphology of the injured skin in the different groups was observed by HE staining and Masson staining. It was found that the tissues in the control mice were normal, whereas the tissues in the model group were damaged with infiltration of inflammatory cells and deposition of collagen (Figure 3B). However, after treatment with miR-26a-5p overexpressed AMSCs-derived exosomes or miR-26a-5p agomir, wound healing was faster with lower infiltration of inflammatory cells, collagen deposition was less, and collagen structure arrangement was thinner and more orderly (Figure 3B). Furthermore, when the miR-26a-5p level was measured, we found that it was significantly reduced in the model mice compared to the control mice (P < 0.05). However, miR-26a-5p was overexpressed in AMSCs-derived exosomes, and miR-26a-5p agomir significantly elevated the miR-26a-5p level caused by damage (*P* < 0.05, Figure 3C). Our *in vivo* experiments confirmed that the AMSCs-derived exosomal miR-26a-5p could facilitate wound healing.

### 3.5 RT-qPCR and Western blot analyses

**FIGURE 4:**
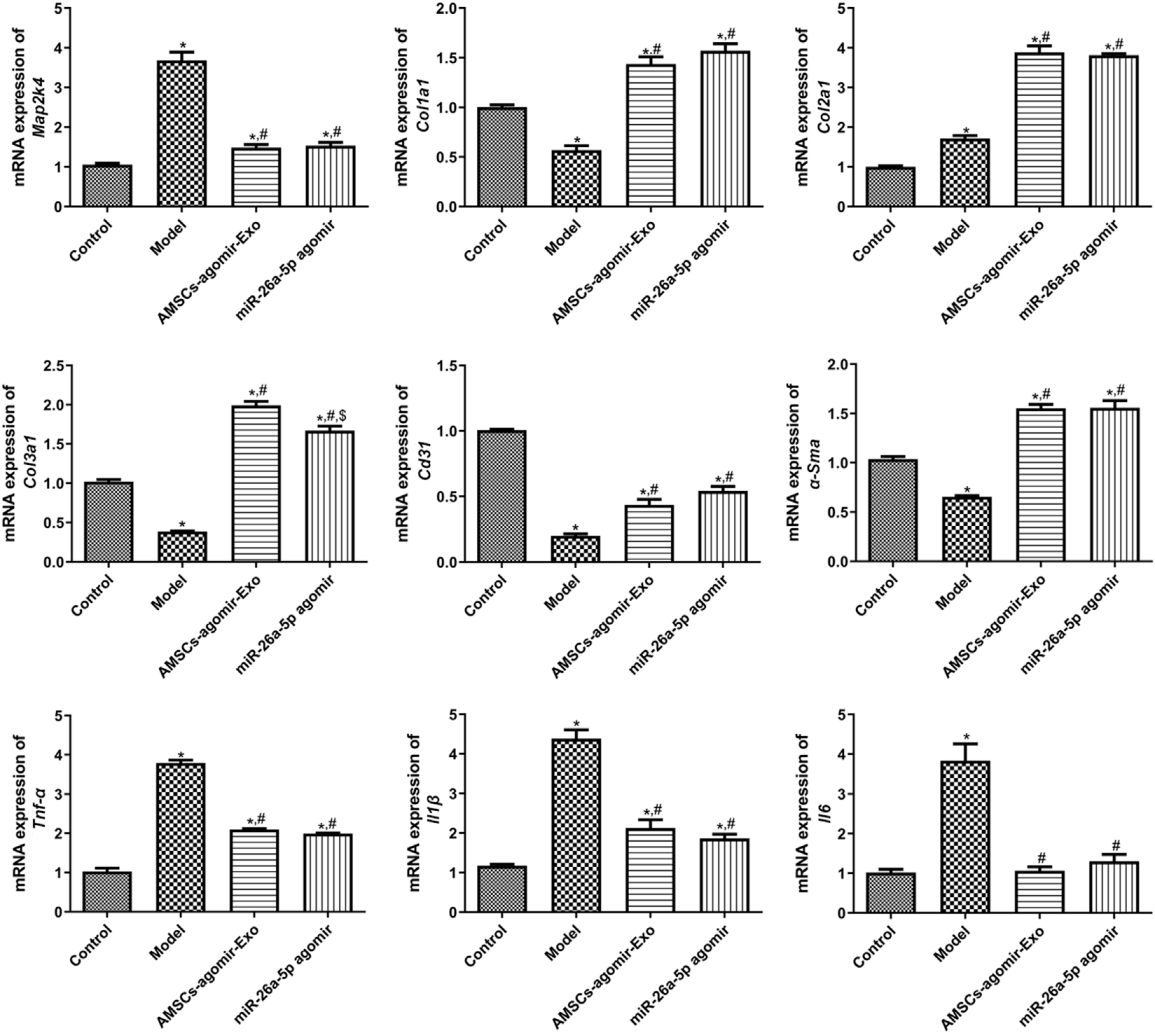
The mRNA expression of related genes, including *Map2k4*, *Col1a1*, *Col2a1*, *Col3a1*, *α-Sma*, *Tnf-α*, *Il1β*, *Il6*, and *Cd31* in the different groups measured by RT-qPCR. N = 3. *: *P* < 0.05, vs. control; ^#^: *P* < 0.05, vs. model; ^$^: vs. AMSCs-agomir-Exo.

**FIGURE 5:**
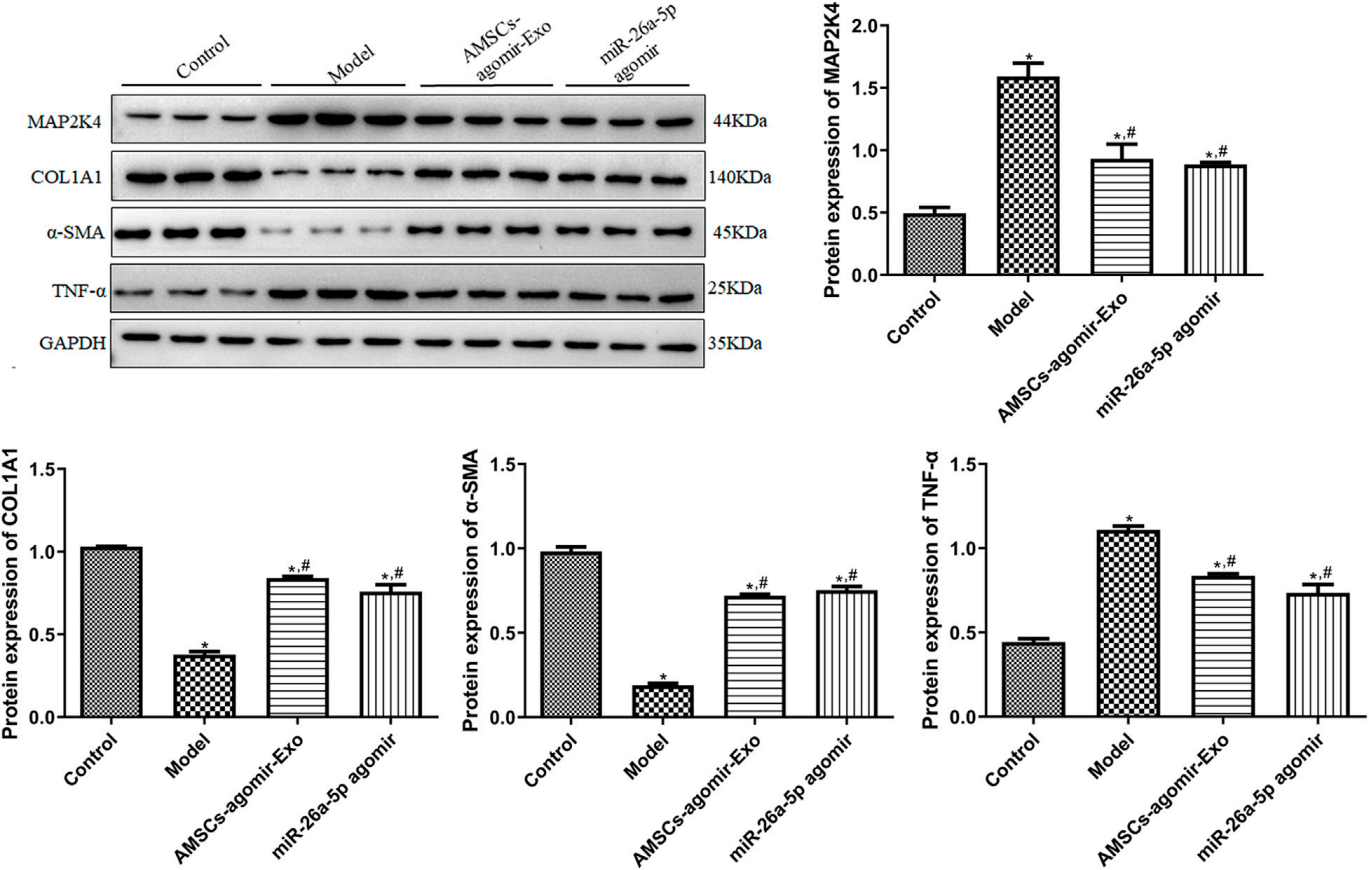
The protein expression of MAP2K4, COL1A1, α-SMA, and TNF-α in the different groups, determined by Western blot. N = 3. *: *P* < 0.05, vs. control; ^#^: *P* < 0.05, vs. model; ^$^: vs. AMSCs-agomir-Exo.

In addition, to unearth the underlying molecular mechanisms of AMSCs-derived exosomal miR-26a-5p promoting wound healing, the expression of some related genes/proteins was detected using RT-qPCR and Western blot. In comparison with the control mice, the target of miR-26a-5p (*Map2k4*) was significantly upregulated in the model mice (*P* < 0.05). However, it was evidently downregulated after treatment with miR-26a-5p-overexpressing AMSCs-derived exosomes and miR-26a-5p agomir compared with the model mice (*P* < 0.05, Figure 4). For *Col1a1*, *Col2a1*, and *Col3a1*, the expression of *Col1a1* and *Col3a1* was evidently downregulated, while *Col2a1* was markedly upregulated in the model mice than the control mice (*P* < 0.05). However, miR-26a-5p-overexpressing AMSCs-derived exosomes and miR-26a-5p agomir significantly upregulated the expression levels of Col1a1, Col2a1, and Col3a1 than the model mice (*P* < 0.05, Figure 4). For *Cd31* and *α-Sma*, their expression was significantly lower in the model mice than in the control mice (P < 0.05); however, it was evidently upregulated in the AMSCs-agomir-Exo and miR-26a-5p agomir groups induced by injury (*P* < 0.05, Figure 4). Afterward, we also measured the expression levels of pro-inflammatory cytokines (TNF-α, IL-1β, and IL-*6*) in the mice with different treatments. The results showed that, compared with the control mice, the expression of Tnf-α, Il1β, and Il6 was significantly upregulated in the model mice (P < 0.05). However, it was evidently downregulated after treatment with miR-26a-5p-overexpressing AMSCs-derived exosomes and miR-26a-5p agomir (*P* < 0.05, Figure 4). Additionally, a Western blot was applied to test the protein expression of MAP2K4, COL1A1, α-SMA, and TNF-α in the different groups. It was evident that the trend of MAP2K4, COL1A1, α-SMA, and TNF-α protein expression in different mice, as determined by Western blot, was consistent with their mRNA expression levels examined by RT-qPCR (Figure 5).

### 3.6 Roles and potential mechanisms of MAP2K4 in wound healing in mice

**FIGURE 6:**
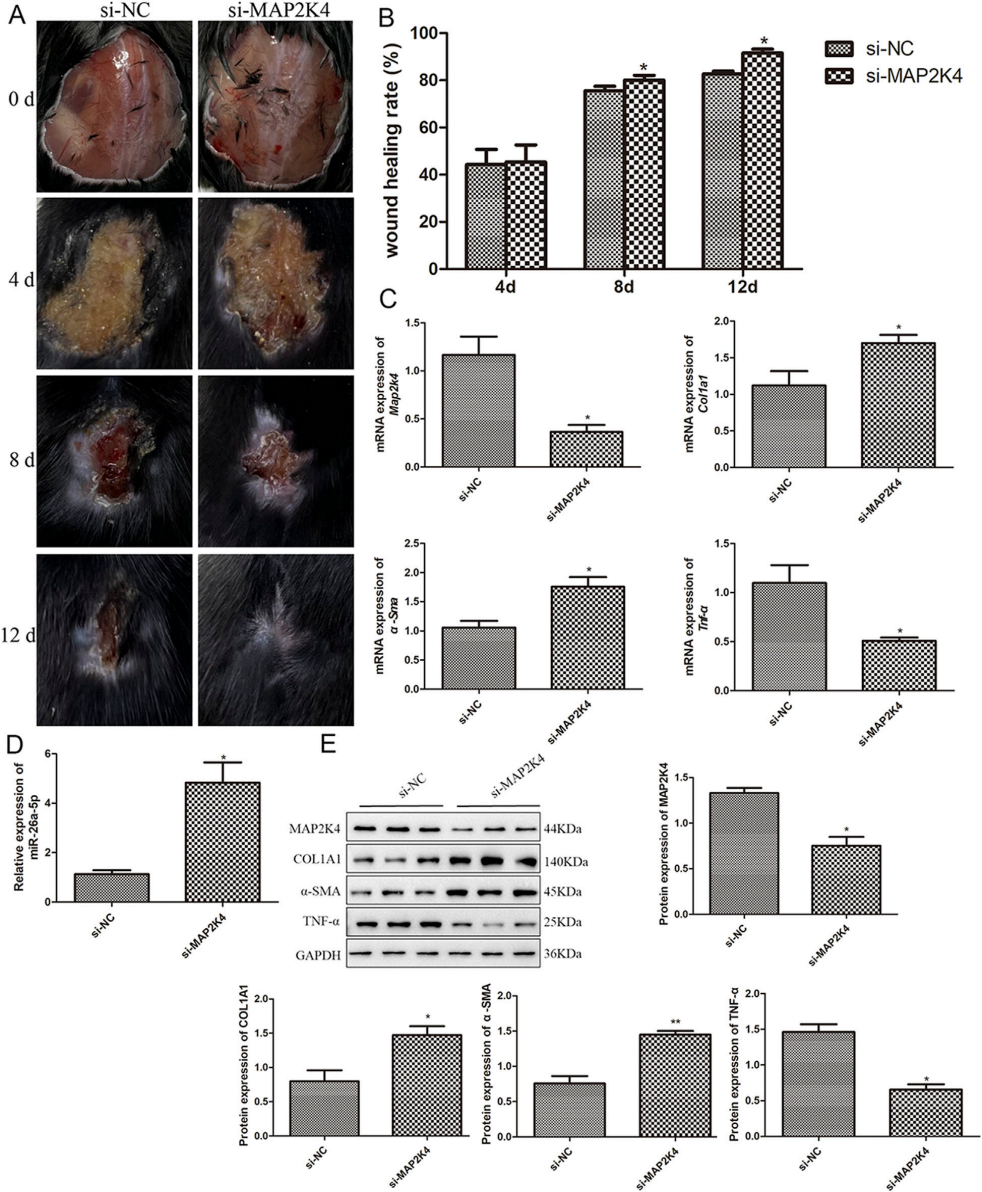
Roles and potential mechanisms of MAP2K4 in wound healing in mice. **(A)** The wound healing process in the skin defect mice treated with si-NC and si-MAP2K4 at days 0, 4, 8, and 12. N = 6. **(B)** Quantification analysis of wound healing rate in the skin defect mice treated with si-NC and si-MAP2K4 at days 4, 8, and 12. N = 6. *: *P* < 0.05, vs. si-NC. **(C)** The mRNA expression of *Map2k4*, *Col1a1*, *α-Sma*, and *Tnf-α* in the skin defect mice treated with si-NC and si-MAP2K4, measured by RT-qPCR. N = 3. *: *P* < 0.05, vs. si-NC. **(D)** The level of miR-26a-5p in the skin tissues of different mice. N = 3. *: *P* < 0.05, vs. si-NC. **(E)** The protein expression of MAP2K4, COL1A1, α-SMA, and TNF-α in the skin defect mice treated with si-NC and si-MAP2K4, detected by Western blot. N = 3. *: *P* < 0.05, vs. si-NC.

Additionally, the roles and potential mechanisms of MAP2K4 in wound healing were investigated. As shown in Figure 6A, with the gradual increase in days, the wounds in the mouse gradually healed in the si-NC and si-MAP2K4 groups. Quantification analysis displayed that at day 4, there was no significant difference in wound healing rate between the si-NC and si-MAP2K4 groups (*P* > 0.05); but at day 8 and 12, the wound healing rate in the si-MAP2K4 group was significantly higher than that in the si-NC group (*P* < 0.05), with the best wound healing rate of the si-MAP2K4 treatment at day 12 (Figure 6B). Then, RT-qPCR showed that compared to the si-NC group, the mRNA expression of *Map2k4* and *Tnf-α* was significantly downregulated in the si-MAP2K4 group (*P* < 0.05); while the mRNA expression of *Col1a1* and *α-Sma* was evidently upregulated (*P* < 0.05, Figure 6C). For miR-26a-5p, its level in the si-MAP2K4 group was significantly elevated compared with the si-NC group (*P* < 0.05, Figure 6D). Finally, the tendency of MAP2K4, COL1A1, α-SMA, and TNF-α protein expression in the different mice, as detected by Western blot, was similar to that determined by RT-qPCR (Figure 6E). These results indicated that MAP2K4 knockdown could promote wound healing, increase the miR-26a-5p level, and upregulate COL1A1 and α-SMA, while downregulating MAP2K4 and TNF-α.

## 4 Discussion

Abnormal wound healing, including chronic wounds (ulcers), scar hyperplasia, and wound dehiscence, can damage normal bodily functions and consume significant resources of the healthcare system (Adebayo et al., 2023). MSCs-derived exosomes are observed to expedite diabetic wound healing by promoting angiogenesis, migration, proliferation, collagen deposition, and ECM remodeling, as well as activating the PI3K/AKT/eNOS pathway (Hu et al., 2021). It has been reported that exosomal miRNAs play pivotal roles in the wound healing process, but the exact mechanisms underlying this are unclear. Therefore, we first analyzed the miRNA profiles 2 days after the injury (in normal control and damaged samples) and identified 13 DEmiRNAs. Their corresponding target genes were enriched in the MAPK cascade, as well as in cAMP, relaxin, Hippo, Apelin, Wnt, and cGMP-PKG signaling pathways.

MAPK is an important intracellular signal that plays a key role in cell response to external stimuli (such as growth factors, cytokines, stress, etc.), and regulates cell proliferation, differentiation, survival, apoptosis, and other physiological processes through phosphorylation of a series of downstream target proteins (Aubé et al., 2024). A previous study demonstrated that NED416, a novel synthetic Sirt1 activator, can accelerate wound closure, macrophage infiltration, and the formation of epithelial and collagen through the MAPK/Rho pathway, thereby promoting wound healing (Wahedi et al., 2020). CAMP is one of the important small molecules present at high concentrations in wound sites, mediating various signaling pathways in stem cell cytoskeleton dynamics, cell adhesion, and migration. This indicates that cAMP is a novel biological factor in tissue repair and regeneration (Peddibhotla et al., 2023). Sang et al. (2024) demonstrated that the loss of lncRNA NEAT1 could facilitate epithelial repair during corneal wound healing by activating the cAMP signaling pathway. Relaxin, a pleiotropic hormone in the insulin family, plays a role not only in promoting cervical softening to facilitate labor but also in regulating collagen renewal, angiogenesis, connective tissue metabolism, and tumor metastasis (Aragón-Herrera et al., 2022). Notably, relaxin could promote bone regeneration through BMP-2-loaded hydroxyapatite microspheres, thereby decreasing the dose of BMP-2 and reducing adverse physiological effects (Injamuri et al., 2020). The Hippo pathway is primarily mediated by YAP and TAZ, and their roles in controlling basal stem cells are evident in epidermal development and skin wound repair (Dey et al., 2020). Nuclear YAP and TAZ localization were observed in the basal layer of the skin, and these nuclear levels were elevated during wound healing (Dey et al., 2020). The Apelin pathway is reported to be involved in alleviating oxidative stress, restoring antioxidant enzyme levels, and reducing cardiovascular, renal, and neurological complications associated with diabetes (Guo et al., 2024). Elabela, a ligand of the apelin receptor, was observed to promote diabetic foot ulcer wound healing by inhibiting TRAF1/NF-κB-mediated inflammation and reducing oxidative DNA damage (Hong et al., 2023). The functions of Wnt pathways have been extensively studied. It is not only involved in the cellular processes (such as proliferation, apoptosis, and cycle), but also in multiple stages of wound healing (Yoon et al., 2023). The cGMP-PKG signaling pathway has been found to enhance keratinocyte migration, as well as play a crucial part in bone remodeling (Kim et al., 2021). Taken together, we speculate that the function of the MAPK cascade and the signaling pathways of cAMP, relaxin, Hippo, Apelin, Wnt, and cGMP-PKG may participate in the process of wound healing; however, their exact roles and underlying mechanisms in wound healing should be further elucidated.

Next, miR-26a-5p was identified as the hub node in the proposed miRNA-mRNA regulatory network, and its target gene, MAP2K4, significantly participated in the MAPK cascade, relaxin signaling pathway, and growth hormone synthesis, secretion, and action, all of which are closely associated with wound healing. Bian et al. (2024) manifested that miR-26a-5p level was lower in diabetic retinopathy (DR) tissues and high glucose-induced Müller cells, and overexpression of miR-26a-5p could improve retinal histopathological injury, and reduce the concentrations of pro-inflammatory cytokines and oxidative stress-related markers in the retina of DR mice by down-regulating *USP14* and inhibiting the activation of NF-κB. Another study revealed that low levels of miR-26a-5p and high levels of RNABP9 were detected in intracerebral hemorrhage tissues and cells. Furthermore, miR-26a-5p overexpression was found to alleviate neuronal apoptosis and brain damage by targeting *RANBP9* (Zhang et al., 2020). MAP2K4 is an upstream member of the MAPK signaling pathway, activating p38 MAPK and JNK, thereby participating in various cellular processes, including proliferation, differentiation, transcriptional regulation, and development (Liu et al., 2019). Our dual luciferase reporter gene assay demonstrated that MAP2K4 was a target of miR-26a-5p. A previous study also employed a dual luciferase reporter gene assay to demonstrate that miR-26a-5p could directly bind to CTGF, and showed that miR-26a-5p could mitigate lung inflammation and apoptosis in lipopolysaccharide-induced acute lung injury by targeting *CTGF* (Li et al., 2021). Therefore, miR-26a-5p/MAP2K4 were selected as the dominating objectives in the follow-up experiments.

Previous research has suggested that human umbilical cord MSCs-derived exosomes can deliver miR-26a-5p to MLE-12 cells, thereby promoting epithelial-mesenchymal transformation in silica-induced pulmonary fibrosis by targeting *ADM17* (Zhao et al., 2023). In addition, AMSCs-derived exosomes have been reported to promote wound healing; however, whether the exosomal miR-26a-5p plays an essential role remains unknown. Therefore, in the current study, exosomes were successfully isolated for the first time from AMSCs with miR-26a-5p overexpression. Thereafter, a skin defect mouse model was established and then administered with exosomes derived from AMSCs with miR-26a-5p overexpression and miR-26a-5p agomir. Based on our results, miR-26a-5p-overexpressing AMSCs-derived exosomes, similar to treatment with miR-26a-5p agomir, could facilitate wound healing, suggesting that AMSCs-derived exosomes may deliver miR-26a-5p to the wound site. Compared with the model mice, AMSCs-derived exosomes delivered miR-26a-5p could downregulate MAP2K4, *Il6*, *Il1β*, and TNF-α, whereas up-regulate COL1A1, *Cd31*, *Col2a1*, α-SMA, and *Col3a1*. Our results have confirmed that MAP2K4 was the target of miR-26a-5p. Liu et al. (2023) indicated that AMSCs-derived exosomal miR-223-3p could target MAPK to regulate the pathways of PI3K-Akt, cAMP, neurotrophin, and cGMP-PKG, thus promoting wound healing. In addition, our results also showed that MAP2K4 knockdown could facilitate wound healing, increase miR-26a-5p levels, and upregulate COL1A1 and α-SMA, while downregulating TNF-α.

Inflammation is the first stage of the wound healing process. It has been reported that inflammation delays wound healing and leads to increased scarring (Hassanshahi et al., 2022). The occurrence and regression of the inflammatory response are major conditions of wound healing, and one of the key factors determining wound quality and healing time (Matar et al., 2023). IL-6, IL-1β, and TNF-α are all pro-inflammatory cytokines that typically influence cell proliferation and migration, immune activation, and ECM remodeling during the wound healing process (Peña and Martin, 2024). Higher concentrations of pro-inflammatory cytokines in cells can lead to an increase in oxidative stress and the deactivation of endothelial and epidermal proliferation, eventually contributing to the formation of permanent wounds (Zhu et al., 2023). Ban et al. (2020) demonstrated that miR-497 overexpression could effectively accelerate diabetic wound closure and reduce the levels of IL-6, IL-1β, and TNF-α.

Fibroblasts are the main cells that synthesize ECM, and their proliferation is a key event in assessing the wound healing cycle (Talbott et al., 2022). COL1A1 and COL3A1 are widely presented in the ECM of various tissues, and COL2A1 is interwoven with other cartilage-specific molecules to build the ECM of cartilages. A previous investigation demonstrated that platelet-rich plasma lysate can protect chondrocytes from synovial-derived inflammatory mediators by upregulating COL1A1, COL2A1, and COL3A1, and downregulating pro-inflammatory cytokines (IL-1β and TNF-α) (Gilbertie et al., 2018). α-SMA is a marker of fibroblast activation, promoting tissue contraction, re-epithelialization, and wound healing (Shinde et al., 2017). The continuous expression of α-SMA in fibroblasts may result in ECM deposition, which is initially protective and plays key roles in wound repair and remodeling (Lee et al., 2019). A recent study by Yang et al. (2024) found that AMSCs-derived exosomes could not only accelerate the healing of diabetic wounds but also improve the healing quality by upregulating α-SMA, COL1A1, and COL3A1, while suppressing Bax/caspase-3. In addition, CD31 is primarily expressed on the surface of endothelial cells and participates in the migration, proliferation, and lumen formation of these cells, thereby promoting the construction of a neovascularization network. Experiments by Xiong et al. (2023) have shown that astragaloside IV-stimulated endothelial progenitor cells-derived exosomes can enhance the wound healing rate, collagen deposition, and increase the number of CD31 and α-SMA positive cells, thereby accelerating wound healing in type 1 diabetes. This literature, together with our findings, suggests that AMSCs-derived exosomes delivering miR-26a-5p may facilitate wound repair by directly down-regulating MAP2K4 and regulating the expression of pro-inflammatory cytokines (IL-6, IL-1β, and TNF-α), ECM-related markers (α-SMA, COL1A1, Col2a1, and Col3a1), and an angiogenesis-related marker (*Cd31*).

Our findings on miR-26a-5p/AMSC-exosome-mediated wound healing extend beyond prior reports (Zhao et al., 2023; Bian et al., 2024) on miR-26a-5p′s regenerative functions by highlighting context-specific mechanisms and translational advantages for cutaneous repair. From a translational perspective, our use of AMSC-derived exosomes further differentiates this work from previous miR-26a-5p studies: compared to hUCMSCs (Zhao et al., 2023), AMSCs are more readily isolated from autologous adipose tissue (e.g., liposuction waste), lowering immune rejection risks for clinical use, and their exosomes inherently contain skin-repair-promoting factors (e.g., growth factors, lipids) that synergize with miR-26a-5p to enhance wound healing, advantages lacking in direct miR-26a-5p administration (Bian et al., 2024), which suffers from poor *in vivo* stability and no such synergism. Our animal experiments further confirm that AMSC-derived exosomal miR-26a-5p exhibits superior wound-healing efficacy compared to free miR-26a-5p agomir, underscoring the value of the carrier. Collectively, while miR-26a-5p′s broad regenerative potential is established, our study is the first to define its role in cutaneous wound healing via targeting MAP2K4, validate AMSCs-derived exosomes as an optimal carrier for miR-26a-5p delivery to skin wounds, and demonstrate its simultaneous regulation of inflammation, angiogenesis, and ECM synthesis, providing a novel mechanistic framework and a more clinically feasible strategy for wound therapy.

However, our study has some limitations. First, a scrambled miRNA-loaded exosome control or naive AMSC-derived exosomes should be included to more comprehensively and accurately demonstrate the specific regulatory role of miR-26a-5p in AMSC-derived exosome-mediated wound healing, and provide more solid experimental evidence for the potential application of AMSC-derived exosomes carrying miR-26a-5p in wound healing therapy. Additionally, the effects of the MAPK cascade and signaling pathways of cAMP, relaxin, Hippo, Apelin, Wnt, and cGMP-PKG in wound healing should be further explored. The roles and underlying mechanisms of other miRNAs in wound healing are also warranted for study *in vitro* and *in vivo*.

In conclusion, through bioinformatics analysis, we identified 13 DEmiRNAs and 143 regulatory target genes that are enriched in the MAPK cascade and signaling pathways of cAMP, relaxin, Hippo, Apelin, Wnt, and cGMP-PKG, which may be involved in the wound healing process. Additionally, *in vivo* experiments demonstrated that AMSCs-derived exosomes carrying miR-26a-5p may expedite the wound healing process by targeting MAP2K4, thereby inhibiting inflammation and promoting angiogenesis and ECM synthesis and deposition. Our findings lay the theoretical foundation for treating various wounds with AMSCs-derived exosomes as carriers, delivering miR-26a-5p and its target gene, MAP2K4, as promising therapeutic targets to promote wound healing.

## Data Availability

The original contributions presented in the study are included in the article/supplementary material, further inquiries can be directed to the corresponding author.
